# Reduced fecal short-chain fatty acids levels and the relationship with gut microbiota in IgA nephropathy

**DOI:** 10.1186/s12882-021-02414-x

**Published:** 2021-06-03

**Authors:** Lingxiong Chai, Qun Luo, Kedan Cai, Kaiyue Wang, Binbin Xu

**Affiliations:** 1 Deparment of Nephrology, Ningbo Hwamei Hospital, University of Chinese Academy of Sciences, No.41, Xibei street, Zhejiang Province 315010 Ningbo, China; 2 Life and Health Industry Research Institute, 315010 Ningbo, Zhejiang Province China

**Keywords:** IgA nephropathy, Short-chain fatty acids, Gut microbiota

## Abstract

**Background:**

IgA nephropathy(IgAN)) is the common pathological type of glomerular diseases. The role of gut microbiota in mediating “gut-IgA nephropathy” has not received sufficient attention in the previous studies. The purpose of this study was to investigate the changes of fecal short-chain fatty acids(SCFAs), a metabolite of the intestinal microbiota, in patients with IgAN and its correlation with intestinal flora and clinical indicators, and to further investigate the role of the gut-renal axis in IgAN.

**Methods:**

There were 29 patients with IgAN and 29 normal control subjects recruited from January 2018 to May 2018. The fresh feces were collected. The fecal SCFAs were measured by gas chromatography/mass spectrometry and gut microbiota was analysed by16S rDNA sequences, followed by estimation of α- and β-diversity. Correlation analysis was performed using the spearman’s correlation test between SCFAs and gut microbiota.

**Results:**

The levels of acetic acid, propionic acid, butyric acid, isobutyric acid and caproic acid in the IgAN patients were significantly reduced compared with control group(*P* < 0.05). Butyric acid(r=-0.336, *P* = 0.010) and isobutyric acid(r=-0.298, *P* = 0.022) were negatively correlated with urea acid; butyric acid(r=-0.316, *P* = 0.016) was negatively correlated with urea nitrogen; caproic acid(r=-0.415,*P* = 0.025) showed negative correlation with 24-h urine protein level.Exemplified by the results of α-diversity and β-diversity, the intestinal flora of IgAN patients was significantly different from that of the control group. Acetic acid was positively associated with *c_Clostridia*(r = 0.357, *P* = 0.008), *o_Clostridiales*(r = 0.357, *P* = 0.008) *and g_Eubacterium_coprostanoligenes_group*(r = 0.283, *P* = 0.036). Butyric acid was positively associated with *g_Alistipes* (r = 0.278, *P* = 0.040). The relative abundance of those were significantly decreased in IgAN group compared to control group.

**Conclusions:**

The levels of fecal SCFAs in the IgAN patients were reduced, and correlated with clinical parameters and gut microbiota, which may be involved in the pathogenesis of IgAN, and this finding may provide a new therapeutic approach.

**Supplementary Information:**

The online version contains supplementary material available at 10.1186/s12882-021-02414-x.

## Introduction

IgA nephropathy(IgAN) is a clinical and pathological syndrome with heterogenous manifestation and progression [1]. It is the most common primary glomerular diseases in the world and an important cause of end stage renal disease (ESRD) in China [2, 3]. The annual incidence of IgAN is about 2.5 people per 100,000, with the highest incidence in East Asia [4], and more than 50 % of patients may develop ESRD after 25–30 years [5]. However, the pathogenesis of IgAN still has not been completely elucidated currently.

More and more studies have confirmed that there is a close correlation between the kidney and gut microecology, called the “gut-kidney axis“ [6, 7]. Recent study has shown that the interaction of microbial, genetic, and dietary factors are thought to induce changes in the function of the intestinal mucosal immune system and promote the progression of IgAN [8].

With the intensive research on gut microecology, the metabolites of gut microbiota are also considered to be important substances involved in the regulation of human vital activities and metabolism. Short-chain fatty acids (SCFAs), including acetic acid, propionic acid, butyric acid and so on, can enter the blood circulation and act as signaling molecules to exert biological effects on peripheral tissues [9, 10]. Patients with chronic kidney disease have a significantly different gut microbiota with decreased levels of SCFAs [11, 12]. Previous study has shown that SCFAs exerts its effects on the “entero-renal axis” mainly through G protein-coupled receptors (GPR) and direct inhibition of histone deacetylase (HDAC) [13]. *In vivo* studies [14, 15], SCFAs, especially acetate and butyrate, were found to inhibit the proliferation of glomerular mesangial cells induced by lipopolysaccharides (LPS) and high glucose in gram-negative bacteria via GPR, and then reversed the production of reactive oxygen species (ROS) and malondialdehyde (MDA) but increased levels of antioxidant enzyme superoxide dismutase (SOD).

There are scarce clinical studies on SCFAs in patients with IgAN. In this study, the changes of fecal SCFAs were measured by gas chromatography mass spectrometry(GC/MS), and their correlations with intestinal flora and clinical indices were investigated to provide new ideas and methods for the role and mechanism of gut-renal axis in IgAN.

## Materials and methods

### Subjects

Twenty-nine patients with IgA nephropathy, 12 males and 17 females, aged 38.21 ± 11.80 years, BMI 23.35 ± 2.88 kg/m^2^, who were followed up at Ningbo Hwamei Hospital of the Chinese Academy of Sciences from January 2018 to May 2018, were enrolled in this study. The tablets they taken during treatment were renin angiotensin inhibitors/angiotensin recetor blockers (28, 96.55 %), calcium channel blockers (5, 17.24 %). A total of 29 age- and sex-matched normal controls were enrolled from Health Check Center, including 12 males and 17 females, aged 38.69 ± 9.90 years, and BMI 23.37 ± 3.46 kg/m^2^ (Supplement Table S1). Inclusion criteria of IgAN group: patients aged ≥ 18 years and diagnosed with IgAN proved by renal biopsy; complete clinical and pathological data; glucocorticoids and/or immunosuppressive agents not taken before renal biopsy. Exclusion criteria: (1) patients diagnosed with secondary IgAN; (2) patients combined with other renal diseases; (3) pregnant or lactating women; (4) patients with infection or stress; (5) patients with acute renal injure or malignant hypertension (diastolic blood pressure ≥ 130 mmHg); (6)patients treated with antibiotics and/or functional food (probiotics and/or prebiotics) in the past three month; (7)patients with type 1 or type 2 diabetes mellitus, neurological or gastro-intestinal diseases; (8) acute myocardial infarction or stroke in the previous six months, severe liver disease, malignancies or with other known immunological or autoimmune disease. All the participants confirmed that there were no remarkable changes in meals and medication for over 1 month. This study was approved by the Ethics Committee of the Ningbo Hwamei Hospital (No.PJ-NBEY-KY-2018-046-01), and the informed consent form was obtained from each subject.

### Clinical data collection

Fasting blood samples were collected from each subject. Urine samples of 24 h were collected from IgAN patients. ADVIA 2400 automatic biochemical analyzer (Siemens, Erlangen, Germany) was used to measure serum creatinine, urea nitrogen, uric acid, albumin, total cholesterol, low-density lipoprotein, triglycerides and 24 h- urine protein. The estimated glomerular filtration rate (eGFR) was calculated by CKD-EPI equation (Levey et al. 2009) [16]. The BC-6800 automatic five-division blood cell analyzer (Mindray, Shenzhen, China) was used to detect white blood cell(WBC) count, neutrophil count, and lymphocyte count,hemoglobin, platelet count, .

### Collection of fecal sample

Fresh fecal samples were collected, quickly placed in ice boxes, and transferred to the laboratory for sub-packaging. After sub-packing, the samples were quickly transferred to a -80 °C cryogenic refrigerator for freezing, and the collection and packaging process was completed within 30 min.

### Fecal SCFA detected by GC/MS

Accurately weigh 50 mg of the fecal sample into a 1.5 ml centrifuge tube, add grinding beads, and then add 300 µL of ultrapure water, and homogenize for 2 min. Centrifuge at 18,000 g for 20 min at 4 °C. Take 200µL of supernatant and 50µL of concentrated sulfuric acid diluted with 50 % water, then add 200ul of ether solution (including internal standard 5 µg/ml), shake for 1 min, then sonicate for 1 min, 4 °C, 12,000 rpm, centrifuge for 20 min, 4 °C, stand for 10 min ether in the upper layer was transferred to an autosampler vial.

Each 1-µL of the sample was injected in split mode with a split ratio 5:1 into an Agilent 7890B gas chromatograph coupled with a Pegasus HT time-of-flight mass spectrometer (Leco Corporation, St. Joseph, MI). Separation was achieved on a DB-FFAP column, with helium(99.9999 %) at a constant flow rate of 1 ml/min. All of these processes were conducted in Metabo-Profile Biotechnology Co.,Ltd. in Shanghai, China.

### Microbiome/metagenome-wide association studies of gut microbiota

DNA was sequenced using 16 S rDNA amplicons. DNA was extracted from fecal samples using the PowerSoil® DNA Isolation Kit (MO BIO). 16 S rDNA/ITS genes of distinct regions (16SV3-V4) were amplified used specific primer with the barcode. All PCR reactions were carried out with Phusion® High-Fidelity PCR Master Mix (New England Biolabs). DNA purity and integrity were analyzed by agarose gel electrophoresis; purified with GeneJET™ Gel Extraction Kit (Thermo Scientif).Sequencing libraries were generated using TruSeq® DNA PCR-Free Sample Preparation Kit (Illumina, USA). The library quality was assessed on the Qubit@ 2.0 Fluorometer (Thermo Scientific). At last, the library was sequenced on an Ion S5™ platform. It was conducted in Zhejiang Tianke High Technology Development Co.Ltd.(Zhejiang, China).

### Statistical analysis

SPSS version 22 (Chicago, IL, US) was used for data analysis. The data were showed as the means ± standard deviations (SD). Student’s t test was used for the continuous data. The continuous non-parametric data were presented as medians (interquartile ranges, IQRs), and were compared using Mann-Whitney U test. Microbial analysis was performed to estimate α-diversity and β-diversity and to compare the differences in microbial community structure between IgAN group and control group. To determine the significantly different taxa between two groups, linear discriminant analysis (LDA) was performed using an online utility [17]. Significantly different bacteria with LDA scores ≥ 2.0 were diagrammed on cladogram. Correlations analysis were performed with spearman’s rank test. Two-tailed p < 0.05 was considered statistically significant.

## Results

### Comparison of clinical parameters between two groups

The age ,gender and BMI data were matched between two groups. Serum creatinine, urea nitrogen and uric acid were increased in IgAN group(*P* < 0.05). The level of eGFR showed no significance between normal control and IgAN groups. Total Cholesterol, triglyceride and LDL levels as well as leukocytes and neutrophils were significantly higher in the IgAN group compared with normal controls (*P* < 0.05). The level of serum albumin was significantly decreased in IgAN group(*P* < 0.05). Lymphocyte, hemoglobin, platelet and, glucose showed no significance between control and IgAN groups (Table 1).

**Table 1:** Comparison of clinical parameters between control and IgAN groups

Clinical parameters	Control group	IgAN group	*P* value
WBC(10^9^/L)	5.69 ± 1.35	6.83 ± 1.78	0.012
Neutrophils(10^9^/L)	3.34 ± 1.24	4.26 ± 1.36	0.014
Lymphocytes(10^9^/L)	1.84 ± 0.44	1.93 ± 0.71	0.549
Hemoglobin(g/L)	140.34 ± 16.64	133.93 ± 17.83	0.137
Platelets(10^9^/L)	230.00 (192.00, 283.50)	230.00 (201.50, 259.00)	0.938
Creatinine (µmol/L)	61.43 ± 13.10	80.29 ± 28.55	0.003
Urea nitrogen (mol/L)	4.63 ± 1.04	5.81 ± 1.82	0.003
Uric acid (µmol/L)	297.57 ± 64.69	370.72 ± 86.03	0.001
Albumin(g/L)	45.35 ± 2.52	40.91 ± 4.59	< 0.001
Total cholesterol(mmol/L)	4.35 ± 0.58	4.93 ± 1.05	0.009
Triglycerides (mmol/L)	1.01 (0.77, 1.33)	1.31 (1.01, 2.18)	0.007
Low-density lipoprotein (mmol/L)	2.51 ± 0.43	2.90 ± 0.77	0.025
Glucose(mmol/L)	4.71 ± 0.43	4.77 ± 0.62	0.580
eGFR(mL/min·1.73 m^2^)	103.89 (99.40,114.34)	101.95 (72.70, 123.44)	0.380
24-h urine protein (mg/24 h)	--	774.75 (366.18, 2030.60)	--

### Fecal short-chain fatty acid levels between two groups

The SCFAs such as acetic acid, propionic acid, butyric acid, isobutyric acid, valeric acid, isovaleric acid, and caproic acid were quantified by GC/MS (Table 2). The levels of acetic acid, propionic acid, butyric acid, isobutyric acid and caproic acid in the IgAN patients were significantly reduced compared with control group(*P* < 0.05).

**Table 2:** Comparison of SCFAs between control and IgAN groups

SCFAs	Control group	IgAN group	*P* value
Acetic acid (mmol/g)	79.56(66.31,135.83)	36.38(30.10, 65.92)	< 0.001
Propionic acid(mmol/g)	6.98(3.37,8.13)	4.16(3.05,5.47)	0.013
Butyric acid(mmol/g)	1.45(1.12,2.14)	0.66(0.49,0.96)	< 0.001
Isobutyric acid(mmol/g)	7.17 ± 5.26	0.68 ± 0.16	< 0.001
Valeric acid(mmol/g)	0.28(0.16,0.36)	0.19(0.09,0.37)	0.129
Isovaleric acid(mmol/g)	0.53(0.39,0.76)	0.39(0.28,0.67)	0.095
Caproic acid(mmol/g)	0.95 ± 0.45	0.54 ± 0.12	< 0.001

### Correlation analysis of fecal SCFAs and clinical parameters

As shown in Table 3, butyric acid(*r*=-0.336, *P* = 0.010) and isobutyric acid(*r*=-0.298, *P* = 0.022) were negatively correlated with urea acid; butyric acid(*r*=-0.316, *P* = 0.016) was negatively correlated with urea nitrogen; caproic acid(r=-0.415, *P* = 0.025) showed negative correlation with 24-h urine protein level. Meanwhile, a strong inverse relationship between SCFA and serum lipids. Propionic acid, butyric acid and isobutyric acid showed negative correlation with total cholesterol (*P* < 0.05). Butyric acid, isobutyric acid and valeric acid showed negative correlation with triglyceridec. Butyric acid and isobutyric acid was negatively correlated with low-density lipoprotein(*P* < 0.05). Interestingly, serum albumin level showed a positive correlation with acetic acid, isobutyric acid and caproic acid(*P* < 0.05).

**Table 3:** Correlations between fecal SCFAs and clinical parameters

Variable 1	Variable 2	r	*P* value
Acetic acid	Albumin	0.337	0.010
Propionic acid	Total cholesterol	-0.264	0.045
Butyric acid	Urea nitrogen	-0.316	0.016
	Low-density lipoprotein	-0.390	0.002
	Total cholesterol	-0.386	0.003
	Uric acid	-0.336	0.010
	Triglyceride	-0.390	0.002
Isobutyric acid	Albumin	0.410	0.001
	Urea nitrogen	-0.299	0.022
	Total cholesterol	-0.306	0.020
	Triglyceride	-0.320	0.014
	Low-density lipoprotein	-0.279	0.034
	Uric acid	-0.298	0.022
Valeric acid	Triglyceride	-0.277	0.036
Caproic acid	Albumin	0.430	0.001
	24-h urine protein	-0.415	0.025

### Characteristics of gut microbiota in IgAN patients

**Fig. 1:**
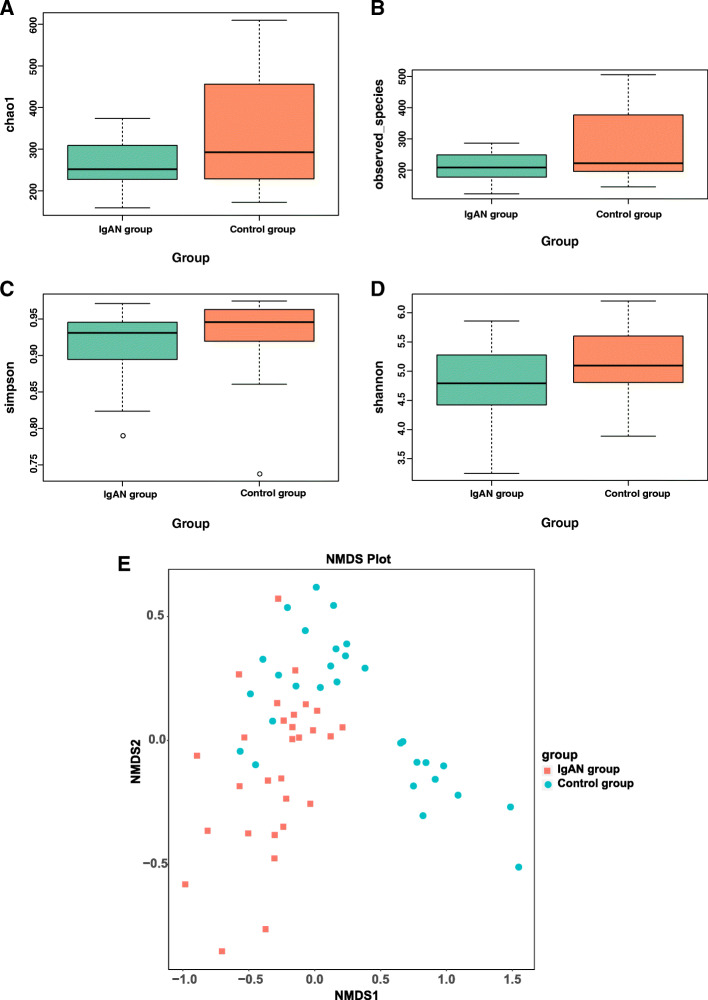
The gut microbiota composition of IgAN patients was significantly different from that of the control. The α-diversity of the microbiota presented as Chao1 (*P* = 0.0945, **A**), observed species (*P* = 0.051, **B**), Simpson (*P* = 0.067, **C**) and the Shannon index (*P* = 0.033, **D**). The β-diversity in the IgAN and control groups was calculated by NMDS (Stress = 0.132) (Fig. 1E)

The α-diversity, including Chao1 (*P* = 0.0945, Fig. 1 A), observed species (*P* = 0.051, Fig. 1B) and Simpson (*P* = 0.067, Fig. 1 C), were approaching to the significant difference between two groups. Shannon index was different between two groups (*P* = 0.033, Fig. 1D). The β-diversity was significantly different between the IgAN and normal control groups by Non-Metric Multi-Dimensional Scaling(NMDS) (Stress = 0.132)(Fig. 1E) .

Overall, 18 bacterial phyla were recovered across the samples. The main phyla were *Firmicutes* (53.40 %), *Bacteroidetes* (36.12 %), *Proteobacteria* (6.89 %), *Actinobacteria* (3.28 %) in IgAN group, the same as those of *Firmicutes* (51.73 %), *Bacteroidetes* (43.55 %), *Proteobacteria* (3.08 %), *Actinobacteria* (0.67 %) in control group. The relative abundance of *Actinobacteria* in IgAN was higher than that in controls with significantly statistical difference(*P* = 0.013).

The LEfSe analysis (Supplementary Figure S1) was used to identify the metagenomic biomarker by way of class comparison. The results of LEfSe showed that 179 bacteria taxa had biologically consistent and statistically significant differences between two groups. The 91 bacteria taxa were more abundant in normal control group, and 88 bacteria taxa were more abundant in IgA nephropathy group.

### Correlations between SCFAs and microbial indexes

**Fig. 2:**
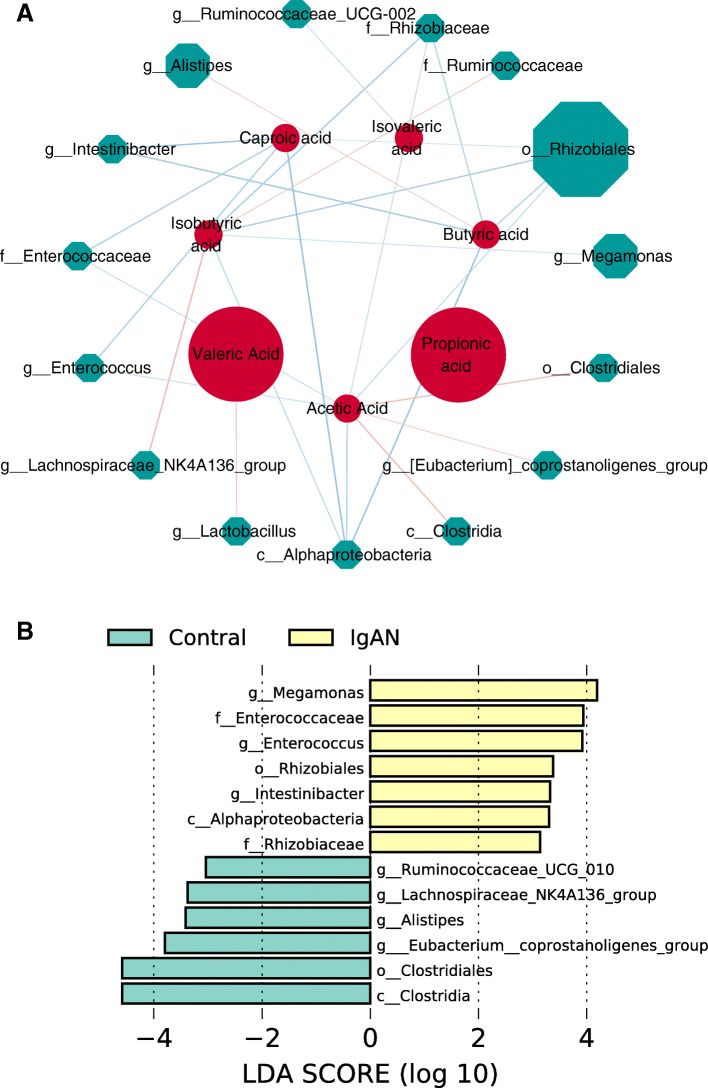
Correlations between SCFAs and microbial indexes. The network diagram of the correlation analysis of microbial indexes and SCFAs (**A**), red represents SCFAs, and green microbial indexes. The color of the edge shows the correlation coefficient (red positive and blue negative)(*P* < 0.05). The node size represents the centrality, that is, the number of consecutive starting from it. LDA was performed to determine the difference of the SCFAs related microbial taxa in two groups (**B**). Significantly different bacteria with LDA scores ≥ 2.0 were diagrammed on cladogram

The correlations of SCFAs and microbial indexes detected in the IgAN patients were analyzed (Fig. 2 A). Acetic acid was positively associated with *c_Clostridia*(*r* = 0.357, *P* = 0.008), *o_Clostridiales*(*r* = 0.357, *P* = 0.008) *and g_Eubacterium_coprostanoligenes_group*(*r* = 0.283, *P* = 0.036). Butyric acid was positively associated with *g_Alistipes* (*r* = 0.278, *P* = 0.040). Isobutyric acid was positively associated with *g_Lachnospiraceae_NK4A136_group* and *f_Ruminococcaceae*(*P* < 0.05). Valeric acid was positively associated with *g_Lactobacillus*(*r* = 0.300, *P* = 0.026). *c_Alphaproteobacteria*, *o__Rhizobiales*, *f_Rhizobiaceae*, *f__Enterococcaceae*, *g__Intestinibacter*, *g__Enterococcus*, *g_Megamonas and g__Ruminococcaceae_UCG-002* were negatively correlated with SCFAs (*P* < 0.05)(Fig. 2 A).

The LDA (Figure F) was used to identify the metagenomic biomarker by way of class comparison. Compared with control group, the relative abundance of *c_Clostridia*, *o_Clostridiales*, *g_Lachnospiraceae_NK4A136_group*, *g_Ruminococcaceae_UCG_010*, *g_Alistipes* and *g_Eubacterium_coprostanoligenes_group were* significantly decreased in the IgAN group. Meanwhile, the relative abundance of *c_Alphaproteobacteria*, *o__Rhizobiales*, *f_Rhizobiaceae*, *f__Enterococcaceae*, *g__Intestinibacter*, *g__Enterococcus*, *g_Megamonas and g__Ruminococcaceae_UCG-002* were increased significantly in the IgAN group(Fig. 2B) .

## Discussion

This study is the first to examine fecal SCFAs in patients with IgAN, and we found significant differences in SCFAs between the IgAN group and normal controls, with significantly lower levels of acetic acid, propionic acid, butyric acid, isobutyric acid and caproic acid in IgAN. There were few studies consistent with our study, where propionic acid and butyric acid levels were significantly reduced in mice with diabetic nephropathy [18]. Wang et al. [19] found significantly lower fecal SCFAs in CKD 5 than in patients with CKD stages 1–4, and negatively correlated with the renal function. Wu et al. [20] found significantly lower serum SCFAs levels in patients with CKD stages 4–5 compared to the normal group.

In our study we detected the fecal microbiota, it is a proxy of the composition of the gut microbiota [21]. There was the dysbiosis of gut microbiota in IgAN patients compared to controls, exemplified by the results of α-diversity, β-diversity and LEfSe analysis in our study. De Angelis et al. [22] reported that the lower microbial diversity was found in IgAN patients estimated by rarefaction, Chao 1and Shannon diversity index. In agreement with our study, Hu X et al. [23] also demonstrated that the community richness of fecal microbiota in the IgAN patients was significantly lower than that in the healthy controls.

The reason for the significant decrease of fecal SCFA in IgAN patients, was related to the decreased relative abundances of SCFA-producing bacteria compared to the normal group. The bacteria taxa of *c_Clostridia*, *o_Clostridiales*, and *g_Eubacterium_coprostanoligenes*_ group were positively correlated with acetic acid, and *g_Alistipes* was positively correlated with butyric acid. Isobutyric acid was positively associated with g_ *Lachnospiraceae_ NK4A136_group* and *f_Ruminococcaceae*. Furthermore, The relative abundance of those were significantly decreased in IgAN group compared to control group. Especially, *Clostridia, Alistipes* and *Ruminococcaceae* were comfirmed as important SCFA-producing bacteria [24, 25]. *Clostridium* was reported to produce acetate by the Wood-Ljungdahl pathway [26]. *Lachnospiraceae* was demonstrated to use lactate and acetate to produce butyrate [27]. Baxter NT et al. [28] found that gut microbiota with an increase in *Ruminococcus* or *Clostridium* were more likely to yield higher butyrate concentrations.

The relative abundance of *c_Alphaproteobacteria*, *o__Rhizobiales*, *f_Rhizobiaceae*, *f__Enterococcaceae*, *g__Intestinibacter*, *g__Enterococcus*, *g_Megamonas and g__Ruminococcaceae_UCG-002* were negatively correlated with SCFAs. SCFAs often exhibited broad-spectrum antimicrobial activity, mainly due to its ability to penetrate and destroy microorganisms. It has been demonstrated that SCFA has both bactericidal and antibacterial functions against oral microorganisms. *In vitro* experiments have demonstrated that butyric acid can inhibit *Salmonella* infection [29].SCFAs are relatively inert to the bacteria that produce them, but can effectively inhibit the growth of other bacteria [30]. In addition to its direct effect on the intestinal barrier, SCFA can lower the pH in the intestinal lumen, which can directly promote the growth of commensal bacteria and inhibit the proliferation of pathogenic bacteria [31].

In this study, the negative correlations between SCFAs and kidney injury-related indicators were found. Butyric acid was negatively correlated with urea acid and urea nitrogen; caproic acid showed negative correlation with 24-h urine protein level. Similar to the findings of previous studies, Wang et al. [19] reported that the serum acetate and butyrate levels in CKD 5 were significantly lower than those in CKD 1–4 patients. Wu et al. [20] found that compared with the normal group, the serum propionic acid level of CKD4-5 patients was significantly lower, and it can be used to distinguish patients with severe renal impairment from the normal group. In the study conducted by Zhang L et al., SCFAs were increased and blood urea nitrogen level was decreased following resistant starch intake [32]. SCFAs exerted its effects on the “entero-renal axis” mainly through G protein-coupled receptors (GPR) and direct inhibition of Histone deacetylase (HDAC) [33]. SCFAs also exerted anti-bacterial, anti-inflammatory and anti-oxidative effects in numerous studies [34].

The role of short-chain fatty acids in IgAN remained unclear. Previous studies have confirmed that SCFAs modulated inflammation both in intestinal and extra-intestinal environments and possessed multifarious effects against inflammatory bowel disease and allergic airway disease by decreasing inflammatory response. [35, 36]. Meanwhile, a strong relationship between IgAN and intestinal inflammation was reported, such as inflammatory bowel diseases [37] and coeliac disease [38]. Qin D et al., found that P-aIgA1 (aggregated IgA1 from IgAN patients) promoted the proliferation of human renal mesangial cells (HMCs), and markedly increased the protein levels of HDAC1 in the cells. Pretreatment with SCFAs inhibitor of HDAC inpartially reversed P-aIgA1-induced cell proliferation and extracellular matrix synthesis in HMCs [39].

Meanwhile, a strong inverse relationship between SCFAs and blood lipids. Propionic acid, butyric acid and isobutyric acid showed negative correlations with total cholesterol (*P* < 0.05). Butyric acid, isobutyric acid and valeric acid showed negative correlation with triglyceridec. Butyric acid and isobutyric acid was negatively correlated with low-density lipoprotein(*P* < 0.05). Previous studies have found that SCFAs also have a role in regulating blood lipids. Propionic acid and butyric acid inhibited isoprenaline- and adenosine deaminase-stimulated lipolysis in the presence of phosphodiesterase (PDE3) inhibitors as well as propionic acid and butyric acid inhibited basal and insulin-stimulated de novo lipogenesis, which was associated with increased phosphorylation, thereby inhibiting the activity of HMG-CoA, the rate-limiting enzyme for fatty acid synthesis. Moreover, SCFAs have an effect on fat storage and mobilization in rat primary adipocytes, thus possibly contributing to healthier adipocyte contracts and improving energy metabolism and reducing circulating free fatty acids [40].

Interestingly, serum albumin level showed a positive correlation with acetic acid, isobutyric acid and caproic acid significantly. Although we didn’t found the report on SCFAs and albumin directly related, there were a few studies on SCFAs and malnutrition [41, 42]. Studies in mouse models revealed that gut-derived SCFAs were actively metabolized and that propionate, butyrate, and acetate played an important role as substrates for glucose metabolism [43]. Recent study [44] presented the potential role of the gut microbiota in anorexia nervosa(AN) by evaluating fecal microbiota composition and SCFAs. Compared to normal-weight participants, the concentrations of butyrate-producing Roseburia spp were reduced in patient with AN.

There are several limitations in this study. First, we found that gut microbiota and SCFAs were involved in IgAN, but the possible mechanisms and pathways have not been elucidated. Second, the sample size was relative small, and the multiple confounders were not controlled for all correlation analyses. Our data should be interpreted with caution, and further studies are needed to verify the role of SCFA produced by the intestinal flora in IgAN.

## Conclusions

The results revealed that fecal SCFAs levels were significantly lower in patients with IgAN, which was related to dysbiosis in IgAN patients. There was a significant correlation between clinical parameters (urea nitrogen, uric acid, proteinuria and lipids ) and SCFAs, which also provides a new basis for future exploration of the role of the entero-renal axis in IgAN.

## Supplementary Information

**Additional file 1:**

**Additional file 2:Supplementary Figure S1**. The Cladogram based on LEfSe results of the IgAN and control groups. The yellow points represent the increased taxa in control group, while the blue points represent the increased taxa in IgAN group.

## Data Availability

The data are available from the corresponding author on reasonable request.
