# Supplementary material for: Reduced fecal short-chain fatty acids levels and the relationship with gut microbiota in IgA nephropathy
Source: BMC Nephrol. 2021 Jun 3;22:209. doi: 10.1186/s12882-021-02414-x (PMC8173972; doi:10.1186/s12882-021-02414-x)
Supplement: Supplementary file 1 — Additional file 1: [file 12882_2021_2414_MOESM1_ESM.doc]

Supplement Table S1 General characteristics of two groups

| General characteristics | Control group | IgAN group | *P* value |
| --- | --- | --- | --- |
| Age (year) | 38.69 ± 9.90 | 38.21±11.80 | 0.862 |
| Male (%) | 12 (41.38) | 12 (41.38) | 1.000 |
| BMI (kg/m^2^) | 23.37±3.46 | 23.35±2.88 | 0.943 |
| ACEIs/ABRs (%) | -- | 28 (96.55) | -- |
| CCBs (%) | -- | 5 (17.24) | -- |

# ACEIs, angiotensin-converting enzyme inbibitors; ARBs, angiotensin II recetor blockers; CCBs, calcium channel blockers.
