# Supplementary figures and images for: Reduced fecal short-chain fatty acids levels and the relationship with gut microbiota in IgA nephropathy
Source: BMC Nephrol. 2021 Jun 3;22:209. doi: 10.1186/s12882-021-02414-x (PMC8173972; doi:10.1186/s12882-021-02414-x)

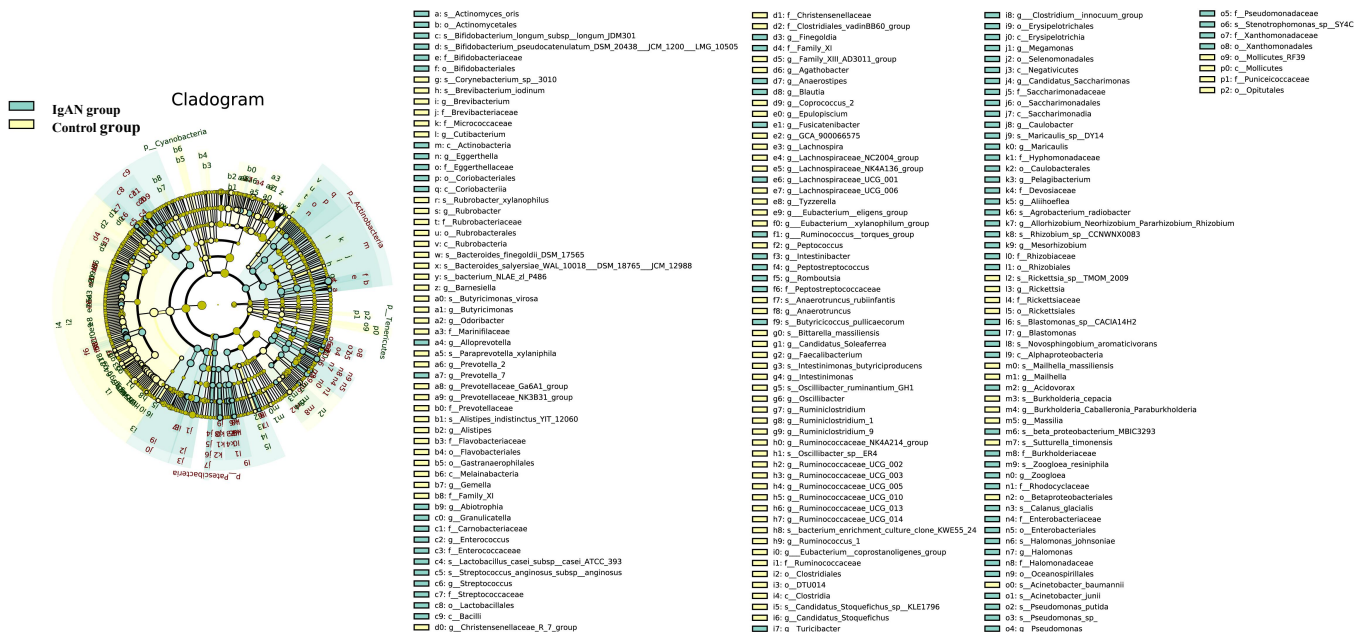

Supplement: Supplementary file 2 — Additional file 2:Supplementary Figure S1. The Cladogram based on LEfSe results of the IgAN and control groups. The yellow points represent the increased taxa in control group, while the blue points represent the increased taxa in IgAN group. [file 12882_2021_2414_MOESM2_ESM.pdf]
